# **α**MSH Blunts Endotoxin-Induced MuRF1 and Atrogin-1 Upregulation in Skeletal Muscle by Modulating NF-**κ**B and Akt/FoxO1 Pathway

**DOI:** 10.1155/2014/179368

**Published:** 2014-09-09

**Authors:** Ana Isabel Martín, Ana Belén Gómez-SanMiguel, Carolina Gómez-Moreira, María Ángeles Villanúa, Asunción López-Calderón

**Affiliations:** Department of Physiology, Faculty of Medicine, Complutense University of Madrid, 28040 Madrid, Spain

## Abstract

Alpha melanocyte stimulating hormone (*α*MSH) has been shown to have anti-inflammatory and anticachectic actions. We hypothesized that *α*MSH administration could attenuate the effect of lipopolysaccharide (LPS) on the skeletal muscle through modifications in IGF-Akt-FoxO1 pathway, or/and in serum corticosterone. Adult male Wistar rats were injected with LPS and/or *α*MSH. *α*MSH administration reduced LPS-induced increase in liver TNF*α* and serum nitrites as well as NF-*κ*B activation in skeletal muscle. In contrast, *α*MSH was not able to prevent the stimulatory effect of LPS on serum concentration of ACTH and corticosterone. LPS decreased serum levels of IGF-I and IGFBP3 and their expression in the liver (*P* < 0.01). However IGFBP3 expression in the gastrocnemius was increased by LPS. Treatment with *α*MSH prevented the effects of LPS on IGFBP3 but not on IGF-I. In the gastrocnemius *α*MSH blocked LPS-induced decrease in pAkt as well as the increase in pNF-*κ*B(p65), FoxO1, atrogin-1, and MuRF1 levels. These results suggest that *α*MSH blunts skeletal muscle response to endotoxin by downregulating atrogenes and FoxO1 at least in part by controlling NF-*κ*B activation and Akt signalling, but not through modifications in the secretion of corticosterone or IGF-I.

## 1. Introduction

Inflammation induces skeletal muscle wasting leading to inflammatory cachexia that causes an increase in morbidity and mortality [1]. Critically ill patients with sepsis have a reduction in muscle mass within the first week [2]. This decrease in muscle mass is secondary to an increase in muscle proteolysis, whereas muscle protein synthesis rate does not seem to be affected [3]. Similarly, experimental sepsis induced by bacterial lipopolysaccharide (LPS) administration increases muscle proteolysis, but it does not decrease protein synthesis [4]. Of the major proteolytic systems, ubiquitin-proteasome pathway is increased, whereas calpain and caspase activities are not changed in sepsis [3]. Between the ubiquitin-proteasome system, two E3 ubiquitin ligases, muscle RING-finger protein-1 (MuRF1) and muscle atrophy F-box (MAFbx/atrogin-1), play an important role in muscle atrophy; they are called atrogenes and serve as early markers of skeletal muscle atrophy, aiding in the diagnosis of muscle disease [5]. Cytokines such as TNF*α* act as potent inducer of the inflammatory response transcription factor NF-*κ*B that increases proteasome-dependent protein breakdown in skeletal muscle [6].

The Forkhead box containing protein O-subclass (FoxOs) are transcription factors that regulate cell proliferation, differentiation, and apoptosis. In their active unphosphorylated state, these proteins reside in the nucleus and promote gene expression of atrophy-stimulating genes such as atrogin-1 and MuRF1 [7]. Skeletal muscle FoxO nuclear localization and transcriptional activities are suppressed by insulin-like growth factor1 (IGF-I)/phosphatidyl inositol 3-kinase (PI3 K)/protein kinase B (Akt) pathway. Phosphorylation of FoxOs by Akt induces translocation from the nucleus and inactivation by degradation in the cytosol. Atrogene induction through FoxO1 and FoxO3a activation is a crucial step in the process leading to muscle atrophy during sepsis [8]. In this sense, sepsis increases FoxO1 mRNA levels as well as nuclear FoxO1 levels and DNA binding activity in gastrocnemius muscle, but not in the heart [9, 10].

Although muscle wasting during sepsis seems to be secondary to multiple factors, the increased release of proinflammatory cytokines and glucocorticoids and the decreased release of IGF-I are important mediators of inflammatory cachexia. Inflammatory stress induced by LPS increases a number of proinflammatory cytokines that are released into the blood stream to activate the innate immune response. LPS administration also has a profound effect on the neuroendocrine system, leading to an increase in glucocorticoid secretion along with a decrease in circulating IGF-I [11–13]. Glucocorticoids are potent mediators of muscle atrophy [14, 15], through atrogin-1 and MuRF1 upregulation [16]. IGF-I is an important regulator of muscle mass; in addition to stimulating muscle protein synthesis through activation of PI3 K and Akt, it also reduces the expression of atrogin-1 and MuRF1 by inhibiting FoxO transcription factors [17, 18]. Taking into account that the IGF-I/Akt pathway is a crucial regulator of muscle mass, the decrease in IGF-I levels together with the increase in serum glucocorticoids can be the mechanism by which sepsis induces muscle wasting.

Alpha melanocyte stimulating hormone (*α*MSH) is a peptide that belongs to the melanocortins family that has many physiological functions, including immune function. *α*MSH has a potent anti-inflammatory activity; it decreases inflammatory mediators such as proinflammatory cytokines and nitric oxide production [19], whereas it increases anti-inflammatory cytokines [20]. It has been shown that the anti-inflammatory action of *α*MSH in a number of cell types is through blockade of NF-*κ*B activation [20–22].

*α*MSH has been shown to ameliorate the course of inflammatory illnesses in experimental animals such as endotoxin-induced hepatitis [23], inflammatory bowel disease [24] and adjuvant-induced arthritis [25]. In addition to its anti-inflammatory activity, we have reported that *α*MSH is also able to prevent arthritis-induced muscle wasting, decreasing MuRF1 and atrogin-1 levels in skeletal muscle [26]. The aim of the present study was to analyze whether peripheral *α*MSH administration is able to prevent endotoxin-induced increase in atrogin-1, MuRF1, and FoxO1 and to analyze the possible role played by the IGF-I system and by endogenous glucocorticoids.

## 2. Material and Methods

### 2.1. *Animals*

All procedures on animals were carried out according to the guidelines recommended by the EU for the care and use of laboratory animals and were approved by the Complutense University Animal Care Committee (approval ID: CEA-UCM 16/12). Experimental design was performed to minimize suffering and the number of animals used. Male Wistar rats weighing 250–275 g were used in all experiments; they were purchased from Charles River Laboratories (Barcelona, Spain). Rats were housed 2-3 per cage, under controlled conditions of temperature (22°C) and light (lights on from 07:30 to 19:30 h). Rats were quarantined for 1 week before any experimental use.

Rats were randomly assigned to the following treatment groups of 10 rats and fed ad libitum: (1) control, i.p. injected with 250 *μ*L sterile saline, (2) control + *α*MSH, i.p. injected with 100 *μ*g/kg *α*MSH trifluoroacetate salt (Bachem, Bubendorf, Switzerland), (3) LPS, i.p. injected with 250 *μ*g/kg LPS (serotype 055:B5, Sigma Chemical Co.), and (4) LPS + *α*MSH, which was simultaneously i.p. injected with both compounds in 250 *μ*L saline. As LPS decreases food intake, a pair-fed (PF) group was added; it was injected with saline and received the same amount of food eaten by the group of rats injected with LPS. Rats received the treatments at 17:00 h and at 08:00 h the following day. This LPS administration protocol was shown to decrease levels of serum IGF-I and its mRNA in the liver [27]. All animals were euthanized by decapitation at 12:00 h, 19 h after the first, and 4 h after the second LPS and/or *α*MSH injection.

Trunk blood was collected and allowed to clot, and the serum was stored at −20°C for IGF-I, IGF binding protein 3 (IGFBP3), adrenocorticotropin hormone (ACTH), corticosterone, and nitrite assays. Spleens were removed, dissected, and weighed; liver and gastrocnemius muscle were removed, frozen immediately in liquid nitrogen, and stored at −80°C for isolation of mRNA and proteins.

### 2.2. RNA Extraction and Real-Time PCR

RNA was extracted using UltraspecTM (Biotecx Laboratories Inc. Houston, Texas, USA). The final concentration of RNA was determined with a BioPhotometer (Eppendorf, Germany), and the integrity of the RNA was confirmed by agarose gel electrophoresis. First-strand cDNA synthesis was performed using 1 *μ*g of total RNA and the Quantiscript Reverse Transcription kit (Qiagen Combh Hilden, Valencia, CA, and USA). Real-time PCR for quantification of mRNA was performed on a SmartCycler (Cepheid, Sunnyvale, CA, USA) using a SYBR-Green protocol on the fluorescence temperature cycler. Each real-time PCR reaction consisted of 2.5 ng cDNA, 1x Takara SYBR Green Premix Ex Taq (Takara BIO INC, Otsu, Shiga, Japan), and 300 nM forward and reverse primers in a reaction volume of 25 *μ*L. Primers for real-time PCR (Table 1) were obtained from Roche (Madrid, Spain) by using the EXIQON Universal Probe Library. The thermal cycling profile consisted of a preincubation step at 95°C for 10 s followed by 40 cycles of 95°C denaturation steps for 15 s, 60°C annealing steps for 30 s, and 72°C extension steps for 30 s. Results were expressed as fold changes in expression of each gene compared with control animals treated with saline using cycle threshold 2(ΔΔCT) method with 18S as reference gene.

### 2.3. Immunoblot

Gastrocnemius was homogenized in RIPA buffer (10 *μ*L/mg) with protease inhibitors cocktail, sodium deoxycholate 12.5 mM, phenylmethane sulfonyl fluoride 100 mM, sodium orthovanadate 12.5 mM, and with phosphatase inhibitors (Sigma-Aldrich, Madrid, Spain) for pAkt/Akt and pFoxO1/FoxO1. Because of the low endogenous levels of total FoxO1 in skeletal muscle, accurate quantification of FoxO1 following nuclear and cytosolic fractionation of whole muscle remains difficult. Therefore, we determined it in the total protein extract. The protein extracts were boiled for 5 min in a 1 : 1 volume of Laemmli loading buffer. Proteins (100 *μ*g) were resolved by electrophoresis on 10–12% polyacrylamide gels under reducing conditions and transferred onto nitrocellulose or polyvinylidene fluoride membranes (Bio-Rad, Madrid, Spain) that were blocked by incubation in 5% nonfat dry milk and 0.1% Tween (Sigma-Aldrich), in Tris-buffered saline. Ponceau-S staining was performed to ensure equal transfer prior to blocking. Membranes were probed overnight at 4°C sequentially with antibodies against pAkt (473), and pFoxO1 (256) (Cell Signalling Technology Inc, Boston, USA), Akt, FoxO1, NF-*κ*Bp65 (C20), pNF-*κ*Bp65 (536) and MuRF1 (Santa Cruz Biotechnology, Santa Cruz, CA, USA) with stripping of membranes, using stripping buffer (Restore Western Blot Stripping Buffer, Thermo-scientific Rockford, II, USA) before each new antibody. Membranes were incubated for 90 min in the appropriate secondary antibody conjugated to horseadish peroxidase (anti mouse IgG, Amersham Biosciences, Little Chalfont, UK; anti rabbit IgG, GE Healthcare, Madrid, Spain; or anti goat IgG, Santa Cruz) and peroxidase activity was detected using enhanced chemiluminescent reagent (Thermo Scientific, Rockford, IL, USA). Band intensities were quantified by densitometry using Gene Tools Analysis software.

### 2.4. Ligand Blot

Serum IGFBP3 levels were measured by ligand blot. Two microlitres of serum were diluted in sample buffer and boiled for 2 min at 90°C and loaded on to 1% SDS-12.5% polyacrylamide gels, and proteins were separated by electrophoresis under nonreducing conditions. Proteins were transferred onto nitrocellulose sheets (HybondTM-C extra, Amersham, UK). The membranes were dried and blocked for 1 h with 5% nonfat dry milk, 0.1% Tween (Sigma-Aldrich), in Tris-buffered saline. Membranes were probed overnight at 4°C with ^125^I-labelled IGF-I (1.5 × 10^6^ cpm/mL). The nitrocellulose sheets were then washed, dried, and exposed at −80°C to X-ray film (Kodak X-Omat AR, Eastman Kodak, Rochester, NY, USA). The film signals were quantified by densitometry using a PC-Image VGA24 program for Windows. The density of the IGFBP3 band in each lane was expressed as the percentage of the mean density of sera from control rats.

### 2.5. IGF-I, ACTH, Corticosterone, and TNF*α* Determinations

Serum IGF-I was measured using the antiserum to human IGF-I (UB2-495), from Dr. Underwood and Dr. Van Wik, and is distributed by the NIDDK Hormone Distribution Program through the National Hormone and Pituitary Program. Levels of IGF-I were expressed in terms of rat IGF-I from Gropep Ltd. (Adelaide, Australia). The intra-assay coefficient of variation was 8%. All samples from the same experiment were run in the same assay.

Serum ACTH and corticosterone was analyzed by a commercial kit from MP Biomedicals, LLC (Orangeburg, NY, USA), following the manufacturer's protocols. Liver TNF*α* was determined by ELISA with a kit from Amersham Biosciences (Barcelona, Spain).

### 2.6. Nitrite Determination

Nitrite and nitrate concentrations in serum were measured by a modified method of Griess assay [28]. Serum was deproteinized to reduce turbidity by centrifugation through a 30 kDa molecular weight filter using a Centrifree Micropartition Device with a YM-30 ultrafiltration membrane (Amicon Division, Millipore Corporation, Bedford, TX, USA), at 15000 rpm for 1 h at 37°C for 300 *μ*L samples. One hundred *μ*L of filtrated serum was mixed with 100 *μ*L of vanadium chloride and was quickly followed by the addition of the Griess reagents. The determination was performed after incubation at 37°C for 30 min. The absorbance was measured at 540 nm. Nitrite and nitrate concentrations were calculated using a NaNO_2_ standard curve.

### 2.7. Statistical Analysis

Statistics were computed using the statistics program STATGRAPHICS plus for Windows. Data are presented as means ± standard error of the mean and were tested with analysis of variance (ANOVA);* post-hoc* comparisons were made using the LSD multiple range test. Statistical significance was set at *P* < 0.05.

## 3. Results 

### 3.1. Anti-Inflammatory Activity of *α*MSH Administration in Rats Injected with LPS

**Figure 1:**
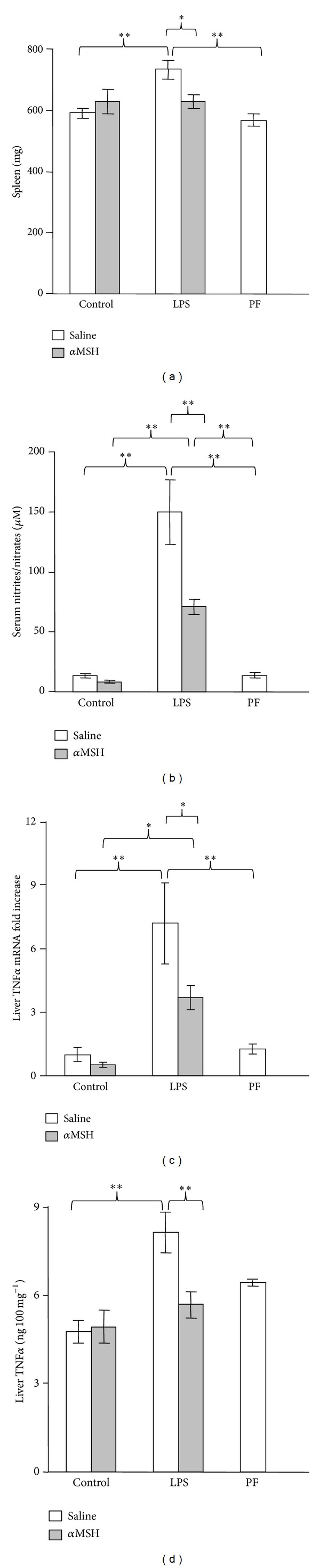
Effect of *α*MSH treatment (100 *μ*g/kg) on spleen weight (a), serum concentrations of nitrites/nitrates (b), liver TNF*α* mRNA (c) and liver TNF*α* protein (d) in control rats or in rats treated with LPS (250 *μ*g/kg). PF = pair-fed rats. *α*MSH treatment decreased the stimulatory effect of LPS administration on spleen weight, serum concentration of nitrites + nitrates, liver TNF*α*, and liver TNF*α* mRNA. Results are expressed as means ± SEM for 6–10 rats per group. ∗∗*P* < 0.01 and ∗*P* < 0.05, LSD multiple comparisons test.

As shown in Figure 1(a), LPS administration increased spleen weight (*P* < 0.01) in rats treated with saline, but not in rats treated with *α*MSH. *α*MSH treatment to control rats did not modify spleen weight. LPS induced a significant increase in serum nitrite/nitrate levels (*P* < 0.01Figure 1(b)). *α*MSH decreased nitrite/nitrate levels (*P* < 0.01) in rats treated with LPS, but not in control rats. Pair-feeding the rats did not modify spleen weight or serum nitrite levels. LPS administration also increased liver TNF*α* as well as its mRNA (*P* < 0.01, Figures 1(c) and 1(d)). In rats treated with *α*MSH, LPS increased liver TNF*α* mRNA (*P* < 0.05) but to lower levels (*P* < 0.05) than those observed in rats injected with saline (Figure 1(c)). However, liver TNF*α* was not increased by LPS administration in rats treated with *α*MSH (Figure 1(d)). Liver TNF*α* mRNA and protein levels were similar in control rats treated with saline and in pair-fed rats.

### 3.2. *α*MSH Ameliorates LPS-Induced Decrease in Food Intake and Body Weight Gain

LPS administration decreased both food intake (*P* < 0.01) and body weight (*P* < 0.01, Table 2). This decrease in body weight gain is not only due to LPS-induced anorexia, since decrease in body weight gain was higher in the rats injected with LPS than in pair-fed rats (*P* < 0.05). In control rats *α*MSH treatment did not modify food intake or body weight gain. However, *α*MSH treatment attenuated the inhibitory effect of LPS on food intake (*P* < 0.05) and on body weight gain (*P* < 0.01).

### 3.3. Serum ACTH and Corticosterone after LPS and/or *α*MSH Administration

**Figure 2:**
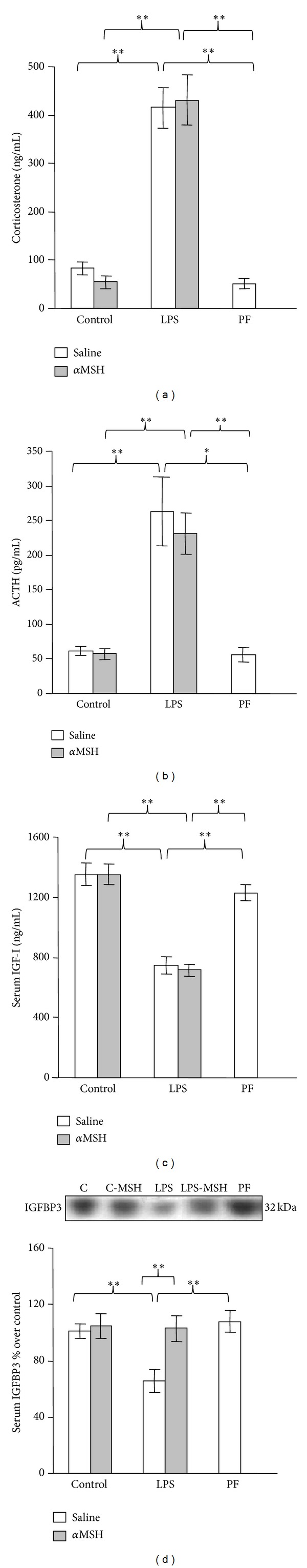
Effect of *α*MSH treatment (100 *μ*g/kg) on serum concentration of corticosterone (a), ACTH (b), IGF-I (c), and IGFBP3 (d) in control rats or in rats treated with LPS (250 *μ*g/kg). PF = pair-fed rats. LPS administration increased the serum concentrations of corticosterone and ACTH (*P* < 0.01), whereas *α*MSH had no significant effect either in control or LPS treated rats. Serum concentrations of IGF1 were decreased by LPS administration in both groups of rats treated with either *α*MSH or saline (*P* < 0.01). LPS treatment decreased serum concentration of IGFBP3 (*P* < 0.01). *α*MSH treatment prevented the effect of LPS on serum IGFBP3. Pair-feeding the rats did not modify serum corticosterone, ACTH, IGF-I, or IGFBP3 levels. Data are expressed as mean ± SEM for *n* = 8–10 rats per group. ∗∗*P* < 0.01 and ∗*P* < 0.05, LSD multiple comparisons test.

Serum levels of corticosterone and ACTH were not significantly modified by pair-feeding the rats or by administering 100 *μ*g/kg *α*MSH to control rats (Figures 2(a) and 2(b)). LPS administration increased serum levels of ACTH and corticosterone (*P* < 0.01) in both groups of rats treated either with saline or with *α*MSH.

### 3.4. *α*MSH Administration Prevents the Effect of LPS on IGFBP3 but Not on IGF-I Levels

In rats treated with saline, LPS decreased circulating levels of IGF-I and IGFBP3 (*P* < 0.01, Figures 2(c) and 2(d)). *α*MSH administration prevented the inhibitory effect of LPS on circulating IGFBP3 levels, whereas it was unable to modify the effect of LPS on serum IGF-I levels. Neither serum concentrations of IGF-I nor IGFBP3 were affected by pair-feeding the rats or *α*MSH administration in control rats.

**Figure 3:**
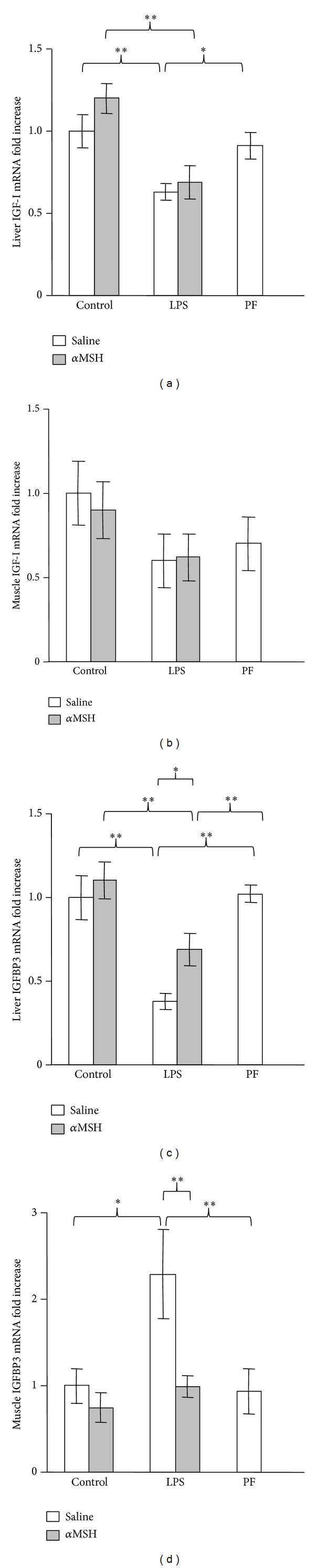
Effect of *α*MSH treatment (100 *μ*g/kg) on IGF-I mRNA in liver (a) and gastrocnemius muscle (b) and on IGFBP3 mRNA in liver (c) and gastrocnemius muscle (d) in control rats or in rats treated with LPS (250 *μ*g/kg). PF = pair-fed rats. Liver IGF-I mRNA was decreased by LPS administration in both groups of rats treated with either *α*MSH or saline (*P* < 0.01). LPS treatment decreased liver IGFBP3 mRNA (*P* < 0.01), but it increased muscle IGFBP3 mRNA (*P* < 0.01). *α*MSH treatment prevented the effect of LPS on muscle IGFBP3 mRNA, whereas it attenuated the inhibitory effect of LPS on liver IGFBP3 mRNA. Pair-feeding the rats did not modify IGF-I or IGFBP3 levels. Data are expressed as mean ± SEM for *n* = 8–10 rats per group. ∗∗*P* < 0.01 and ∗*P* < 0.05, LSD multiple comparisons test.

As observed in circulating IGF-I and IGFBP3, LPS administration decreased IGF-I and IGFBP3 expression in the liver (*P* < 0.01, Figures 3(a) and 3(c)). Treatment with *α*MSH did not modify the inhibitory effect of LPS on liver IGF-I mRNA. In contrast, *α*MSH attenuated LPS-induced decrease in liver IGFBP3, where the rats treated with LPS and *α*MSH had IGFBP3 mRNA levels between those of control rats and of the rats treated with LPS alone. Pair-feeding the rats or *α*MSH administration to control rats did not modify liver expression of IGF-I or IGFBP3.

Figures 3(b) and 3(d) show IGF-I and IGFBP3 expression in the gastrocnemius muscle. LPS had a different effect on IGF-I and IGFBP3 mRNA in the gastrocnemius than in the liver. Gastrocnemius IGF-I mRNA was not significantly affected by LPS, *α*MSH, or by pair-feeding the rats (Figure 3(b)). In contrast, LPS administration increased IGFBP3 mRNA in the gastrocnemius muscle (*P* < 0.05). *α*MSH treatment prevented the stimulatory effect of LPS on muscle IGFBP3, whereas it did not modify IGFBP3 mRNA in control rats.

### 3.5. *α*MSH Administration Prevents the Effect of LPS on NF-*κ*B(p65), pAkt, FoxO1, MuRF1, and Atrogin-1 in the Gastrocnemius Muscle

**Figure 4:**
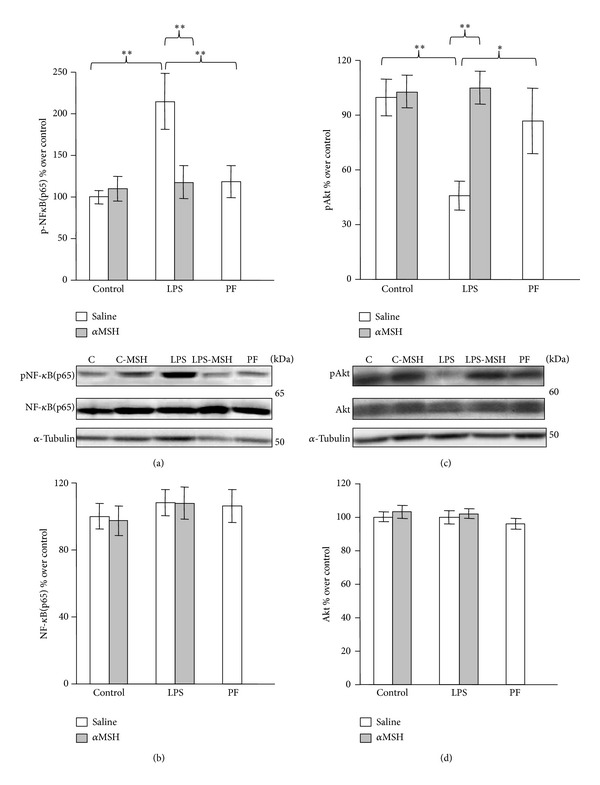
PhosphoNF-*κ*B(p65) (a), NF-*κ*B(p65) (b), phospho-Akt (c), and total-Akt (d) in gastrocnemius muscle of control, LPS and pair-fed (PF) rats treated with *α*MSH (100 *μ*g/kg) or saline. LPS increased phosphoNF-*κ*B(p65) (*P* < 0.01) and decreased pAkt (*P* < 0.01) in the rats treated with saline but not in rats treated with *α*MSH. Data represent mean ± SEM (*n* = 7–10 rats). ∗∗*P* < 0.01 and ∗*P* < 0.05, LSD multiple comparisons test.

The effects of LPS and *α*MSH administration on NF-*κ*B(p65) and Akt in the gastrocnemius muscle are shown in Figures 4(a), 4(b), 4(c), and 4(d). LPS increased phosphoNF-*κ*B(p65) in the rats treated with saline (*P* < 0.01, Figure 4(a)) but not in those treated with *α*MSH. There were no modifications in gastrocnemius NF-*κ*B(p65) levels in none of the experimental groups (Figure 4(b)). LPS had a negative effect on Akt signalling, since it decreased phosphoAkt levels (*P* < 0.01, Figure 4(c)). *α*MSH treatment blocked the inhibitory effect of LPS on Akt phosphorylation. Neither *α*MSH nor pair-feeding the rats modified pAkt levels in the gastrocnemius muscle. All experimental groups had similar total Akt levels (Figure 4(d)).

**Figure 5:**
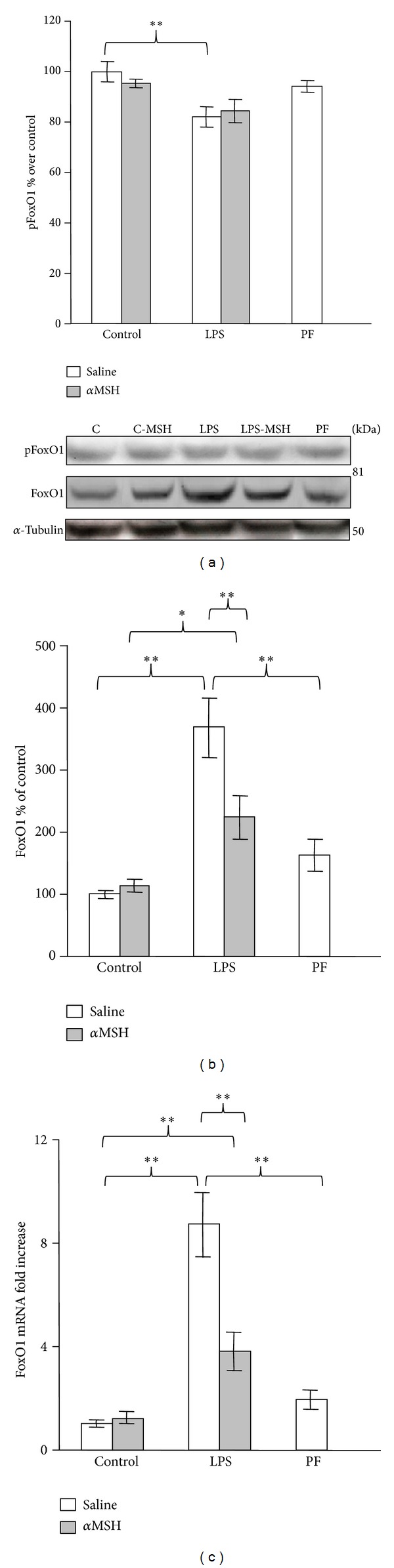
PhosphoFoxO1 (a), total FoxO1 (b), and FoxO1 mRNA levels (c) in gastrocnemius muscle of control, LPS and pair-fed (PF) rats treated with *α*MSH (100 *μ*g/kg) or saline. LPS decreased pFoxO1 (*P* < 0.05), whereas it increased FoxO1 and FoxO1 mRNA (*P* < 0.01) in rats treated with saline. *α*MSH administration prevented LPS-induced increase in FoxO1 and its mRNA. Data represent mean ± SEM (*n* = 8–10 rats). ∗∗*P* < 0.01 and ∗*P* < 0.05, LSD multiple comparison test.

LPS-induced decrease in muscle pAkt levels was associated with an increase in the muscle content of the active transcription factor FoxO1, as well as in its mRNA levels (*P* < 0.01, Figures 5(b) and 5(c)). *α*MSH treatment prevented the stimulatory effect of LPS on FoxO1 and FoxO1mRNA levels, since the rats treated with LPS and *α*MSH had lower FoxO1 content than the rats injected with LPS and saline and content similar to the levels of pair-fed rats. Pair-feeding the rats increased mean FoxO1 and its mRNA levels in the muscle, but this increase was not significant. LPS administration decreased pFoxO1 levels (*P* < 0.05, Figure 5(a)). LPS also decreased pFoxO1 levels in the rats treated with *α*MSH, but the decrease was not significant in comparison to the levels of control rats treated with *α*MSH.

**Figure 6:**
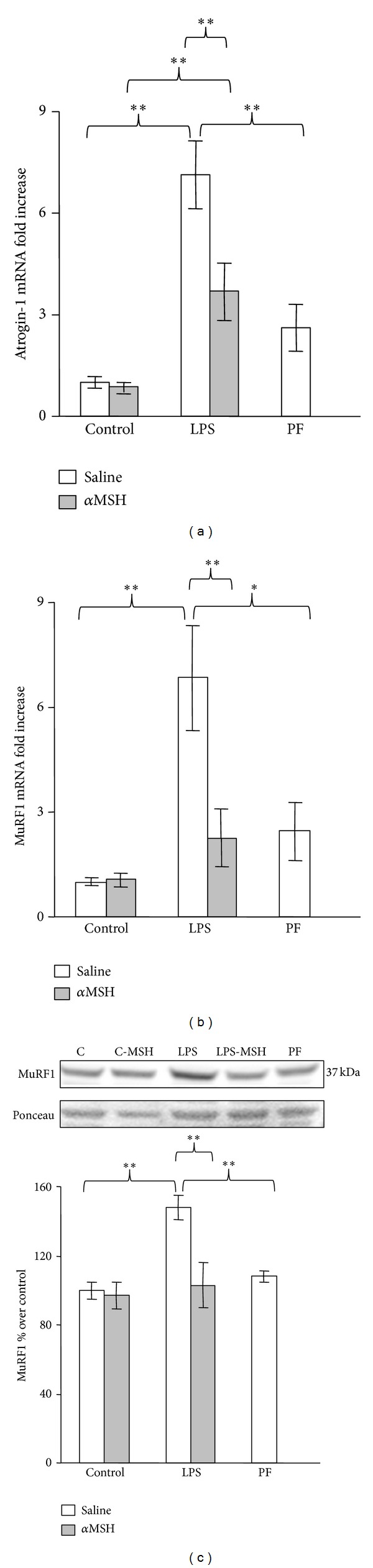
Atrogin-1 mRNA (a), MuRF1 mRNA (b), and MuRF1 (c) in gastrocnemius muscle of control, LPS and pair-fed (PF) rats treated with *α*MSH (100 *μ*g/kg) or saline. LPS increased atrogin-1, MuRF1, and MuRF1 mRNA levels (*P* < 0.01), and *α*MSH administration prevented those increases. Data represent mean ± SEM (*n* = 7–10 rats). ∗∗*P* < 0.01 and ∗*P* < 0.05, LSD multiple comparison test.

Atrogenes expression coincides with the activity of the NF-*κ*B and the Akt/FoxO1 pathway in the five groups of rats. Atrogin-1 mRNA was increased by LPS administration (*P* < 0.01, Figure 6(a)). Rats treated with both LPS and *α*MSH had lower gastrocnemius atrogin-1 mRNA than rats that received LPS alone (*P* < 0.01), and similar to pair-fed rats. LPS administration also increased muscle MuRF1 and its mRNA expression in the gastrocnemius muscle (*P* < 0.01, Figures 4(b) and 4(c)). *α*MSH treatment blocked LPS-induced increase in MuRF1 and in its mRNA in the gastrocnemius muscle.

## 4. Discussion

Our data show that *α*MSH treatment was not able to modify the stimulatory effect of LPS on circulating ACTH and corticosterone or the inhibitory effect of LPS on circulating and liver IGF-I. However, *α*MSH treatment induced anti-inflammatory changes in liver and muscle and prevented negative effects of LPS on skeletal muscle, such as inhibition of Akt phosphorylation and increase in IGFBP3 expression and in FoxO1, atrogin-1, and MuRF1 levels in the gastrocnemius muscle.

As previously reported [19, 20] *α*MSH prevented the increase in liver TNF*α* after LPS injection. The anti-inflammatory effect of *α*MSH was also evidenced by the ability of this hormone to decrease LPS-induced increase in spleen weight and serum concentration of nitrites/nitrates and to prevent NF-*κ*B phosphorylation in muscle after LPS administration.

In accordance with data we have previously reported in arthritic rats [26], peripheral *α*MSH treatment attenuated LPS-induced anorexia and the decrease in body weight gain. On the contrary, intracerebroventricular administration of *α*MSH potentiates LPS-induced reduction in food intake during 6 h [29]. Those data and the fact that systemic *α*MSH treatment did not decrease food intake in control rats (present data and [26]) suggest that the ability of systemic *α*MSH to cross the blood brain barrier is low, as it has previously been reported [30], and the effects we observed are exerted at peripheral level.

In spite of its anti-inflammatory effect, peripheral administration of *α*MSH was not able to decrease the stimulatory effect of LPS administration on the adrenal axis. However, when centrally administered, *α*MSH has been reported to decrease the stimulatory effect of LPS on serum corticosterone levels, at a very low dosage [31, 32]. In addition, *α*MSH is not able to prevent the stimulatory effect of CRH on pituitary ACTH, suggesting a hypothalamic rather than pituitary site of action of *α*MSH on the hypothalamic-pituitary-adrenal axis [32].

In contrast to our data, an inhibitory effect of peripheral *α*MSH administration on LPS-induced increase in ACTH has been reported in mice [33]. Discrepancies can be due to the fact that these authors used a higher dosage of *α*MSH. We tested a higher dosage of *α*MSH (200 *μ*g/kg), and again peripheral *α*MSH administration was not able to prevent the effect of LPS on circulating IGF-I, ACTH, and corticosterone (author's unpublished observation). Another possibility is that *α*MSH decreases the response to LPS within the first few minutes after administration. In this sense, Huang et al. [34] reported that in rats, 100 *μ*g/kg *α*MSH partially blocks the stimulatory effect of LPS, since it prevents the increase in plasma corticosterone 60 min but not 30 or 120 min after LPS administration. Nevertheless, we cannot exclude that at a higher dosage peripheral administration of *α*MSH may be able to prevent the stimulatory effect of LPS on corticosterone levels.

As previously reported [11, 12, 27], LPS administration decreased circulating IGF-I and IGBP3 and their gene expression in the liver. Similar data have also recently been reported in septic patients [35]. LPS decreases serum IGF-I, acting both in the liver and at a central level [36, 37]. The effects of *α*MSH treatment on serum levels of IGF-I and IGFBP3 in rats injected with LPS were different, since *α*MSH was not able to modify the inhibitory effect of LPS on IGF-I, whereas it prevented the LPS-induced decrease in serum IGFBP3 and its expression in the liver. The different responses of both proteins to *α*MSH treatment may be due to the fact that they are regulated differently [38–40]. In addition, they are produced by different liver cells, whereas IGF-I is mainly produced in hepatocytes, IGFBP3 expression is only found in nonparenchymal cells [38, 41]. The effect of *α*MSH on liver IGFBP3 can be exerted directly, since liver cells express all *α*MSH receptors (MCRs) and their expression is upregulated during the acute phase response [42]. In addition, *α*MSH inhibits endotoxin-induced upregulation of the acute-phase cytokines (interleukin1 (IL1), IL6, and TNF*α*) in isolated Kupffer cells [42]. Therefore, *α*MSH can prevent LPS-induced decrease in IGFBP3 synthesis by nonparenchymal cells, although it is not able to prevent the decrease in IGF-I expression.

In contrast to the effect of LPS on liver IGFBP3 expression, local expression of IGBP3 was increased in skeletal muscle by LPS administration. These data indicate that regulation of IGFBP3 varies depending on the tissue. In chronic inflammation induced by arthritis we have found that IGFBP3 expression is also increased in the gastrocnemius muscle but not in the liver [43]. An increase in muscle IGFBP3 has also been reported 2 days after muscle injury, during the early phase of regeneration when muscle is invaded by inflammatory cells, especially by macrophages [44]. In peripheral tissues local IGFBP3 is a well-documented inhibitor of cell growth and/or promoter of apoptosis by a non-IGF-dependent mechanism (for review see [45]). IGFBP3 is produced by myogenic cell cultures and it suppresses proliferation in an IGF-dependent and -independent manner [46]. Accordingly, the increased expression of IGFBP3 in skeletal muscle can contribute to inflammation-induced muscle wasting. As in the case of circulating levels of IGFBP3, *α*MSH treatment was able to prevent LPS-induced increase in muscle IGFBP3 expression.

In the gastrocnemius muscle LPS decreased pAkt, whereas it increased pNF-*κ*B(p65) and FoxO1 active protein and its mRNA. These data are in accordance with those previously reported by other authors [9, 47, 48]. As expected, the decrease in pAkt and the increase in pNF-*κ*B and FoxO1 protein were associated with an increased expression in both atrogenes atrogin-1 and MuRF1. Treatment with *α*MSH prevented the effects of LPS on the muscle: the decrease in Akt phosphorylation and the increase in NF-*κ*B(p65) phosphorylation and FoxO1, MuRF1, and atrogin-1, reaching levels similar to those found in pair-fed rats. All these data indicate that *α*MSH administration blocks LPS-induced alterations in Akt/FoxO1 signalling and downstream gene targets of FoxO1, atrogin-1, and MuRF1 in gastrocnemius muscle. Similarly, in arthritic rats, *α*MSH administration prevents upregulation of both atrogin-1 and MuRF1 [26].

Taking into account that *α*MSH treatment prevented the inhibitory effect of LPS on pAkt in the gastrocnemius, we expected that *α*MSH would be able to modify the inhibitory effect of LPS on IGF-I. However, *α*MSH administration was unable to prevent LPS-induced decrease in serum and liver IGF-I expression. The possibility exists that normalization of Akt activity in gastrocnemius of the rats treated with LPS and *α*MSH is related to normalization of NF-*κ*B activity and/or to muscle IGFBP3 levels. There are several data indicating that IGFBP3 can play an inhibitory role in the PI3K/Akt signalling pathway in different types of cancer cells, through an IGF-independent effect [49, 50]. IGFBP3 also decreases Akt phosphorylation in noncancer cell cultures such as adipocytes [51].

Although *α*MSH did not modify the effect of LPS on the main hormones related to muscle wasting (IGF-I and corticosterone), it had anti-inflammatory actions, and in the skeletal muscle it prevented the decrease in pAkt levels, the activation of NF-*κ*B and FoxO1, and the upregulation of atrogin-1 and MuRF1. Because we analyzed the acute effects of LPS treatment, it was not possible to measure muscle atrophy. However, in arthritic rats chronic *α*MSH treatment decreases both muscle wasting and inflammation [26], in spite of not preventing the increase in serum concentrations of corticosterone and ACTH or the decrease in serum concentration of IGF-I (authors' unpublished observation). Another treatment that shows anti-inflammatory and anticachectic effects in arthritic rats is the PPAR-*α* agonist fenofibrate. Fenofibrate administration decreases skeletal muscle atrophy [52], but it is also unable to prevent the effects of arthritis on serum IGF-I and corticosterone levels [43].

It has been postulated that stimulation of muscle proteolysis requires two events, increased circulating glucocorticoid and/or impaired insulin signalling [53]. PI3K/Akt pathway, which had previously been shown to be sufficient to induce hypertrophy via activation of protein synthesis pathways, can also dominantly suppress the activation of atrophy pathways, determined by the induction of atrophy markers MuRF1 and atrogin-1 [54]. IGF-I, activated PI3K, or activated Akt is sufficient to inhibit the upregulation of MuRF1 and atrogin-1 induced by the glucocorticoid dexamethasone [18]. In our data, the preventive effect of *α*MSH on LPS-induced increase in atrogin-1 and MuRF1 levels can be explained by the normalization of NF-*κ*B and Akt/FoxO1 pathways. This can be secondary to a reduced release of cytokines, as reflected by the normalization of pNF-*κ*B levels, and/or to the normalization of IGFBP3 levels in the gastrocnemius.

## 5. Conclusion

Our data suggest that in rats injected with LPS, *α*MSH exerts anti-inflammatory and antiproteolytic activities in skeletal muscle downregulating FoxO1 and atrogene activation at least in part by controlling NF-*κ*B and Akt activation. These results support *α*MSH as a novel potential therapeutic agent for clinical use in patients with sepsis that show a reduction in muscle mass. The pathways through which *α*-MSH blunts muscle wasting should be clarified by future studies analyzing the possible melanocortin receptors involved.

## Figures and Tables

**Table 1:** Primers for real-time PCR.

Gene	Forward primer (5′ to 3′)	Reverse primer (5′ to 3′)	Product bp
18S	GGTGCATGGCCGTTCTTA	TCGTTCGTTATCGGAATTAACC	60
TNF*α*	TGAACTTCGGGGTGATCG	GGGCTTGTCACTCGAGTTTT	122
FoxO1	TCAGGCTAGGAGTTAGTGAGCA	GGGGTGAAGGGCATCTTT	95
Atrogin-1	GAACAGCAAAACCAAAACTCAGTA	GCTCCTTAGTACTCCCTTTGTGAA	74
MuRF1	TGTCTGGAGGTCGTTTCCG	ATGCCGGTCCATGATCACTT	58
IGF-I	GCTATGGCTCCAGCATTCG	TCCGGAAGCAACACTCATCC	62
IGFBP-3	GGAAAGACGACGTGCATTG	GCGTATTTGAGCTCCACGTT	78

**Table 2:** Effect of 0.1 mg/kg *α*MSH administration on body weight gain and food intake in control rats or in rats treated with 250 *μ*g/kg LPS. PF = pair-fed rats. *α*MSH treatment attenuated the inhibitory effect of LPS administration on body weight gain and food intake. Results are expressed as means ± SEM for 10 rats per group. Values without the same letter are significantly different. LSD multiple comparisons test.

	Body weight gain g/19 h	Food intake g/19 h
Control-saline	4.3 ± 0.6^a^	18.6 ± 0.35^a^
Control-*α*MSH	6.1 ± 0.7^a^	20.3 ± 0.45^a^
LPS-saline	−21.7 ± 0.7^b^	3.9 ± 0.67^b^
LPS-*α*MSH	−14.3 ± 3.6^c^	6.5 ± 1.4^c^
PF	−14.7 ± 1.2^c^	3.9

## References

[1] Callahan LA, Supinski GS (2009). Sepsis-induced myopathy. *Critical Care Medicine*.

[2] Poulsen JB, Møller K, Jensen CV, Weisdorf S, Kehlet H, Perner A (2011). Effect of transcutaneous electrical muscle stimulation on muscle volume in patients with septic shock. *Critical Care Medicine*.

[3] Klaude M, Mori M, Tjäder I, Gustafsson T, Wernerman J, Rooyackers O (2012). Protein metabolism and gene expression in skeletal muscle of critically ill patients with sepsis. *Clinical Science*.

[4] Kovarik M, Muthny T, Sispera L, Holecek M (2012). The dose-dependent effects of endotoxin on protein metabolism in two types of rat skeletal muscle. *Journal of Physiology and Biochemistry*.

[5] Lecker SH, Jagoe RT, Gilbert A (2004). Multiple types of skeletal muscle atrophy involve a common program of changes in gene expression. *FASEB Journal*.

[6] Pijet B, Pijet M, Litwiniuk A, Gajewska M, Pajak B, Orzechowski A (2013). TNF-*α* and IFN-s-dependent muscle decay is linked to NF-*κ*B- and STAT-1*α*-stimulated Atrogin1 and MuRF1 genes in C2C12 myotubes. *Mediators of Inflammation*.

[7] Sandri M, Sandri C, Gilbert A (2004). Foxo transcription factors induce the atrophy-related ubiquitin ligase atrogin-1 and cause skeletal muscle atrophy. *Cell*.

[8] Reed SA, Sandesara PB, Senf SM, Judge AR (2012). Inhibition of FoxO transcriptional activity prevents muscle fiber atrophy during cachexia and induces hypertrophy. *FASEB Journal*.

[9] Nystrom GJ, Lang CH (2008). Sepsis and AMPK activation by AICAR differentially regulate FoxO-1, -3 and -4 mRNA in striated muscle. *International Journal of Clinical and Experimental Medicine*.

[10] Smith IJ, Alamdari N, O'Neal P, Gonnella P, Aversa Z, Hasselgren P-O (2010). Sepsis increases the expression and activity of the transcription factor Forkhead Box O 1 (FOXO1) in skeletal muscle by a glucocorticoid-dependent mechanism. *International Journal of Biochemistry and Cell Biology*.

[11] Fan J, Char D, Kolasa AJ (1995). Alterations in hepatic production and peripheral clearance of IGF-I after endotoxin. *The American Journal of Physiology*.

[12] Soto L, Martín AI, Millán S, Vara E, López-Calderón A (1998). Effects of endotoxin lipopolysaccharide administration on the somatotropic axis. *Journal of Endocrinology*.

[13] Priego T, Ibáñez de Cáceres I, Martín AI, Villanúa MA, López-Calderón A, Ibáñez de I (2002). Glucocorticoids are not necessary for the inhibitory effect of endotoxic shock on serum IGF-I and hepatic IGF-I mRNA. *Journal of Endocrinology*.

[14] Hasselgren P-O, Alamdari N, Aversa Z, Gonnella P, Smith IJ, Tizio S (2010). Corticosteroids and muscle wasting: role of transcription factors, nuclear cofactors, and hyperacetylation. *Current Opinion in Clinical Nutrition and Metabolic Care*.

[15] Schakman O, Kalista S, Barbé C, Loumaye A, Thissen JP (2013). Glucocorticoid-induced skeletal muscle atrophy. *International Journal of Biochemistry and Cell Biology*.

[16] Castillero E, Alamdari N, Lecker SH, Hasselgren P-O (2013). Suppression of atrogin-1 and MuRF1 prevents dexamethasone-induced atrophy of cultured myotubes. *Metabolism: Clinical and Experimental*.

[17] Sacheck JM, Ohtsuka A, McLary SC, Goldberg AL (2004). IGF-I stimulates muscle growth by suppressing protein breakdown and expression of atrophy-related ubiquitin ligases, atrogin-1 and MuRF1. *The American Journal of Physiology—Endocrinology and Metabolism*.

[18] Stitt TN, Drujan D, Clarke BA (2004). The IGF-1/PI3K/Akt pathway prevents expression of muscle atrophy-induced ubiquitin ligases by inhibiting FOXO transcription factors. *Molecular Cell*.

[19] Gonindard C, Goigoux C, Hollande E, D'Hinterland LD (1996). The administration of an *α*-MSH analogue reduces the serum release of IL-1*α* and TNF*α* induced by the injection of a sublethal dose of lipopolysaccharides in the BALB/c mouse. *Pigment Cell Research*.

[20] Brzoska T, Luger TA, Maaser C, Abels C, Böhm M (2008). Alpha-melanocyte-stimulating hormone and related tripeptides: biochemistry, antiinflammatory and protective effects in vitro and in vivo, and future perspectives for the treatment of immune-mediated inflammatory diseases. *Endocrine Reviews*.

[21] Manna SK, Aggarwal BB (1998). *α*-melanocyte-stimulating hormone inhibits the nuclear transcription factor NF-*κ*B activation induced by various inflammatory agents. *The Journal of Immunology*.

[22] Lee S-N, Ryu J-H, Joo J-H (2011). *α*-Melanocyte-stimulating hormone inhibits tumor necrosis factor *α*-stimulated MUC5AC expression in human nasal epithelial cells. *The American Journal of Respiratory Cell and Molecular Biology*.

[23] Chiao H, Foster S, Thomas R, Lipton J, Star RA (1996). *α*-melanocyte-stimulating hormone reduces endotoxin-induced liver inflammation. *Journal of Clinical Investigation*.

[24] Rajora N, Boccoli G, Catania A, Lipton JM (1997). *α*-MSH modulates experimental inflammatory bowel disease. *Peptides*.

[25] Ceriani G, Diaz J, Murphree S, Catania A, Lipton JM (1994). The neuropeptide alpha-melanocyte-stimulating hormone inhibits experimental arthritis in rats. *Neuroimmunomodulation*.

[26] Gómez-SanMiguel AB, Martín AI, Nieto-Bona MP (2013). Systemic *α*-melanocyte-stimulating hormone administration decreases arthritis-induced anorexia and muscle wasting. *The American Journal of Physiology: Regulatory Integrative and Comparative Physiology*.

[27] Granado M, Martín AI, Priego T, Villanúa MA, López-Calderón A (2006). Inactivation of Kupffer cells by gadolinium administration prevents lipopolysaccharide-induced decrease in liver insulin-like growth factor-I and IGF-binding protein-3 gene expression. *Journal of Endocrinology*.

[28] Miranda KM, Espey MG, Wink DA (2001). A rapid, simple spectrophotometric method for simultaneous detection of nitrate and nitrite. *Nitric Oxide—Biology and Chemistry*.

[29] Huang Q-H, Hruby VJ, Tatro JB (1999). Role of central melanocortins in endotoxin-induced anorexia. *The American Journal of Physiology—Regulatory Integrative and Comparative Physiology*.

[30] Wilson JF (1988). Low permeability of the blood-brain barrier to nanomolar concentrations of immunoreactive alpha-melanotropin. *Psychopharmacology*.

[31] Caruso C, Mohn C, Karara AL (2004). Alpha-melanocyte-stimulating hormone through melanocortin-4 receptor inhibits nitric oxide synthase and cyclooxygenase expression in the hypothalamus of male rats. *Neuroendocrinology*.

[32] Cragnolini AB, Perelló M, Schiöth HB, Scimonelli TN (2004). *α*-MSH and *γ*-MSH inhibit IL-1*β* induced activation of the hypothalamic-pituitary-adrenal axis through central melanocortin receptors. *Regulatory Peptides*.

[33] Rivier C, Chizzonite R, Vale W (1989). In the mouse, the activation of the hypothalamic-pituitary-adrenal axis by a lipopolysaccharide (endotoxin) is mediated through interleukin-1. *Endocrinology*.

[34] Huang Q-H, Hruby VJ, Tatro JB (1998). Systemic *α*-MSH suppresses LPS fever via central melanocortin receptors independently of its suppression of corticosterone and IL-6 release. *American Journal of Physiology—Regulatory Integrative and Comparative Physiology*.

[35] Papastathi C, Mavrommatis A, Mentzelopoulos S, Konstandelou E, Alevizaki M, Zakynthinos S (2013). Insulin-like Growth Factor I and its binding protein 3 in sepsis. *Growth Hormone and IGF Research*.

[36] Priego T, Granado M, Ibáñez de Cáceres I, Martín AI, Villanúa MA, López-Calderón A (2003). Endotoxin at low doses stimulates pituitary GH whereas it decreases IGF-I and IGF-binding protein-3 in rats. *Journal of Endocrinology*.

[37] Priego T, Ibáñez de Cáceres I, Martín AI, Villanúa MA, López-Calderón A (2005). Endotoxin administration increases hypothalamic somatostatin mRNA through nitric oxide release. *Regulatory Peptides*.

[38] Lelbach A, Scharf J-G, Ramadori G (2001). Regulation of insulin-like growth factor-I and of insulin-like growth factor binding protein-1, -3 and -4 in cocultures of rat hepatocytes and Kupffer cells by interleukin-6. *Journal of Hepatology*.

[39] Priego T, Ibáñez de Cáceres I, Martín AI, Villanúa MA, López-Calderón A (2003). Endotoxin decreases serum IGFBP-3 and liver IGFBP-3 mRNA: comparison between Lewis and Wistar rats. *Molecular and Cellular Endocrinology*.

[40] Martín AI, López-Menduiña M, Castillero E, Granado M, Villanúa MA, López-Calderón A (2008). Cyclooxygenase-2 activation by endotoxin mediates the decrease in IGF1, but not in IGFBP3, gene expression in the liver. *Journal of Endocrinology*.

[41] Zimmermann EM, Li L, Hoyt EC, Pucilowska JB, Lichtman S, Lund PK (2000). Cell-specific localization of insulin-like growth factor binding protein mRNAs in rat liver. *American Journal of Physiology: Gastrointestinal and Liver Physiology*.

[42] Malik IA, Triebel J, Posselt J (2012). Melanocortin receptors in rat liver cells: change of gene expression and intracellular localization during acute-phase response. *Histochemistry and Cell Biology*.

[43] Castillero E, López-Menduiña M, Martín AI, Villanúa MA, López-Calderón A (2011). Comparison of the effects of the n-3 polyunsaturated fatty acid eicosapentaenoic and fenofibrate on the inhibitory effect of arthritis on IGF1. *Journal of Endocrinology*.

[44] Jennische E, Hall CM (2000). Expression and localisation of IGF-binding protein mRNAs in regenerating rat skeletal muscle. *APMIS*.

[45] Jogie-Brahim S, Feldman D, Oh Y (2009). Unraveling insulin-like growth factor binding protein-3 actions in human disease. *Endocrine Reviews*.

[46] Pampusch MS, Kamanga-Sollo E, White ME, Hathaway MR, Dayton WR (2003). Effect of recombinant porcine IGF-binding protein-3 on proliferation of embryonic porcine myogenic cell cultures in the presence and absence of IGF-1. *Journal of Endocrinology*.

[47] Crossland H, Constantin-Teodosiu D, Gardiner SM, Constantin D, Greenhaff PL (2008). A potential role for Akt/FOXO signalling in both protein loss and the impairment of muscle carbohydrate oxidation during sepsis in rodent skeletal muscle. *The Journal of Physiology*.

[48] Schakman O, Dehoux M, Bouchuari S (2012). Role of IGF-I and the TNF*α*/NF-*κ*B pathway in the induction of muscle atrogenes by acute inflammation. *The American Journal of Physiology—Endocrinology and Metabolism*.

[49] Alami N, Page V, Yu Q (2008). Recombinant human insulin-like growth factor-binding protein 3 inhibits tumor growth and targets the Akt pathway in lung and colon cancer models. *Growth Hormone and IGF Research*.

[50] Cortés-Sempere M, De Miguel MP, Pernía O (2013). IGFBP-3 methylation-derived deficiency mediates the resistance to cisplatin through the activation of the IGFIR/Akt pathway in non-small cell lung cancer. *Oncogene*.

[51] Chan SSY, Twigg SM, Firth SM, Baxter RC (2005). Insulin-like growth factor binding protein-3 leads to insulin resistance in adipocytes. *Journal of Clinical Endocrinology & Metabolism*.

[52] Castillero E, Nieto-Bona MP, Fernández-Galaz C (2011). Fenofibrate, a PPAR*α* agonist, decreases atrogenes and myostatin expression and improves arthritis-induced skeletal muscle atrophy. *American Journal of Physiology—Endocrinology and Metabolism*.

[53] Hu Z, Wang H, In HL, Du J, Mitch WE (2009). Endogenous glucocorticoids and impaired insulin signaling are both required to stimulate muscle wasting under pathophysiological conditions in mice. *The Journal of Clinical Investigation*.

[54] Bodine SC, Stitt TN, Gonzalez M (2001). Akt/mTOR pathway is a crucial regulator of skeletal muscle hypertrophy and can prevent muscle atrophy in vivo. *Nature Cell Biology*.

